# Determination of the sources and antimicrobial resistance patterns of *Salmonella* isolated from the poultry industry in Southern Ethiopia

**DOI:** 10.1186/s12879-017-2437-2

**Published:** 2017-05-18

**Authors:** Reta Duguma Abdi, Fisseha Mengstie, Ashenafi Feyisa Beyi, Takele Beyene, Hika Waktole, Bedasso Mammo, Dinka Ayana, Fufa Abunna

**Affiliations:** 1 College of Veterinary Medicine and Agriculture, Addis Ababa University, P.O. Box 34, Bishoftu, Oromia Ethiopia; 2 Department of Animal Science, Institute of Agriculture, University of Tennessee Knoxville, Knoxville, TN USA; 3 Southern Agricultural Research Institute, Bonga Agricultural Research Center, P.O.Box 101, Bonga, Ethiopia; 4 Department of Animal Sciences, University of Florida, Gainesville, Florida, USA

**Keywords:** Antimicrobial resistance, Biosecurity, Multiplication centers, Risk factors, Poultry, *Salmonella* prevalence

## Abstract

**Background:**

Ethiopia set an ambitious masterplan to increase chicken meat and egg production from 2015 to 2020. Poultry breeding, multiplication and distribution centers in the country have received executive order to import, amplify and distribute commercial chickens to end users. The biosecurity and the pathogen fauna of the centers have not been evaluated as to whether the centers could implement the mission effectively without any risk. Thus, the aim of this study was to evaluate the biosecurity practices and the pathogen prevalence, risk factors and their antimicrobial resistance (AMR) using *Salmonella* as case study.

**Methods:**

Routine farm workers of the centers were interviewed about the different management (biosecurity) practices using a checklist. Samples (*n* = 270) from different sources consisting of chicken’s cloacal swab (*n* = 244), personnel hand swab (*n* = 9) and bedding (*n* = 17) were collected from three chicken multiplication centers. Standard bacteriological methods were used for the isolation of *Salmonella*. Disk diffusion method was used for drug sensitivity testing.

**Results:**

Antimicrobials were often over prescribed without confirming the cause of ill health and without susceptibility testing. The general biosecurity and flock management practices were substandard. *Salmonella* was isolated from 45 (16.7%) of the 270 samples. Its prevalence was significantly (p<0.05) associated with location of the multiplication center, 27% at Bonga and 10.6% at Hawassa. Sample type was also significantly (p<0.05) affected in that it was higher in the bedding (35.3%) and personnel hand swabs (33.3%) than in the chicken cloaca (14.8%), which demonstrates the poor biosecurity and personnel hygienic practices in the centers. All of the 45 isolates (100%) exhibited resistance to kanamycin and sulfamethoxazole-trimethoprim, nalidixic acid (97.8%), ampicillin (97.8%), cefoxitin (97.8%), streptomycin (97.8%) tetracycline (97.8%), chloramphenicol (91.3%), ciprofloxacin (31.1%), and gentamicin (0%). Alarmingly, 42 isolates (93.4%) exhibited multidrug resistance (MDR) to ≥ 8 drugs and all 45 isolates had resistance to ≥ 3 drugs. The high rate of *Salmonella* isolation from (i) bedding, (ii) personnel hand swabs (iii) chickens, (iv) presence of more MDR isolates, (v) coupled with poor biosecurity practices in the centers could pose a risk for spreading of pathogens and drug resistant genes to the smallholder chicken producers and the public.

**Conclusions:**

We conclude that the poultry breeding, multiplication and distribution centers in Ethiopia, as they stand currently, seem to be a source of pathogens and AMR isolates at least for *Salmonella*. Therefore, strict biosecurity, personnel safety, prudent drug use, regular monitoring and traceability of *Salmonella* serotypes or genotypes and AMR are recommended.

## Background

Ethiopia has about 56.87 million chickens comprising mainly indigenous chickens, with the majority (95%) kept in low-input low-out-put village chicken production systems [1, 2]. Chicken provides an important source of income in addition to offering eggs and meat for the poor smallholder households [1]. However, the 0.5 kg annual per capita egg consumption in Ethiopia is five times lower than the average 2.3 kg per capita for other sub-Sahara African countries [3]. The Ethiopian indigenous chickens cannot be produced and fill the national demand for chicken meat and eggs despite the fact that they harbor suitable phenotypic traits for ecological, social and cultural context of Ethiopia [1, 4]. Rhode Island Red, Australorp, New Hampshire and White Leghorns chickens were imported to Haromaya, Jimma, Bishoftu and Shashemene in 1950s [5]. This led to the establishment of modern poultry farms in 1959 able to distribute commercial chickens to urban producers. Shortly thereafter, seven poultry multiplication and distribution centers were established in different locations under the Ministry of Agriculture to distribute commercial chickens [6]. Although Ethiopia started utilization of imported high producing commercial chicken’s years ago, the supply to fill the demand has never been met, even though the number of multiplication centers has increased to 15 [7]. Poultry multiplication centers are defined as those government owned centers engaged in importation of improved commercial chickens from developed countries, rear, multiply and distribute chicks of mostly up to 3 month old all over the country. The centers are not for profit as chicks are sold with around 50% below real market prices unlike the private commercial poultry farms [7]. However, the poultry sub-sector has remained at subsistence level. The national demand for chicken products remains still under-met, and hence malnutrition and poverty is widespread. Thus, Ethiopia has launched recently a master plan for livestock development. The plan aims to increase chicken meat production by 247% and egg production by 827% in five years, 2020 [8].

Improved commercial laying hens, which produce about 325 eggs per year and broilers that can reach 2.5 kg in 42 days with high feed efficiency and product quality are available elsewhere [9]. These animals are targeted for importation and incorporation in the current Ethiopian livestock development master plan based on preliminary experiences on these animals in the country [10–12].

In Ethiopia, farm hygiene, biosecurity, diagnostic capabilities, monitoring, tracing and notification of pathogens and AMR at the farm and national level are not well developed [7, 13] and uncontrolled antimicrobial use and misuse is common [14]. Thus, the burden of pathogens in poultry multiplication centers is poorly known in Ethiopia, which may entail risk of distributing infected chickens unless appropriately handled.

One of the major pathogens devastating the poultry industry is *Salmonella* [15]. Salmonellosis causes severe economic damage to chicken production by reducing production, 100% morbidity, and 20% mortality in affected flocks [15]. Furthermore, the vertical and horizontal transmission ability of *Salmonella* complicates the spread and control of the disease; hence, it remains a problem on the farm for prolonged time [16]. *Salmonella* is also known as one of the major food-borne zoonotic pathogens. In United State, it is responsible for 1.4 million cases of human illness, 20,000 hospitalizations and over 500 deaths annually [17]. The ability of *Salmonella* to acquire antimicrobial resistance (AMR) is another capability of this pathogen rendering it difficult to eliminate. The emergence and spread of such resistant strains among food animals is life threatening and a global public health concern, as they are often non-treatable with currently available antimicrobials [18]. Animal agriculture such as poultry farming and antibiotic usage on the farms are a hot debate topic, because the overuse may be a contributing factor for the entrance of AMR pathogens and AMR genes to the food chain [19]. Thus, *Salmonella* in poultry remains a political issue due to its impact on food production, animal welfare, public health and antimicrobial resistance.

A study in northern Ethiopia reported that the prevalence of *Salmonella* in the local and commercial breeds was 39.3% and 29.2%, respectively [20], 42.7% in indoor chickens and 40.8% in free ranging in western Oromia at Jimma [21]. The limited studies have reported the presence of multidrug resistant *Salmonella* strains in chicken farms [22, 23], which cannot be killed by antimicrobials and hence increase the morbidity, mortality and costs associated disease. Overall, studies conducted on *Salmonella* in Ethiopia are scarce. It is difficult to understand the real picture in the country because of the diverse chicken population growing in the different agro-ecologies of the country [1, 4]. Considerable differences in level of susceptibility to *Salmonella* have been reported among different chicken breeds [24]. Moreover, controlling *Salmonella* in poultry operations is complicated because there are numerous potential sources of contamination, including chicks, feed, rodents, wild birds, equipment and personnel in the poultry processing plant [25] as well as hatcheries [26]. One of the key areas for controlling *Salmonella* is to know its source and dissemination pathways. Ethiopia has developed research-extension programs that interact to transfer improved technology packages from agricultural research centers (e.g. commercial chicken breeding, multiplication and distribution centers) to end users [27]. However, little is known about where *Salmonella* establishes at the poultry breeding, multiplication and/or distribution centers in Ethiopia. Therefore, the aim of this study was to (i) identify poor farm practices in biosecurity that trigger pathogen flare-ups, (ii) determine the prevalence and (iii) AMR patterns of *Salmonella* isolates in three poultry breeding, multiplication and distribution centers in Southern Ethiopia.

## Methods

### Description of study areas

This study was conducted in Hawassa and Bonga, Southern Nations, Nationalities and Peoples Region (SNNPR), from November 2014 to May 2015. Hawassa is the capital of the SNNPR and is located at 275 km south of Addis Ababa. The annual rainfall and temperature of the area varies from 800 mm to 1000 mm and 20 °C to 25 °C, respectively. Bonga is an administrative city for Kaffa zone and is situated at 460 km south-west of Addis Ababa. The mean annual rainfall in the area is about 1710 mm, and the monthly temperature varies from 18 °C to 21 °C [2, 28].

### Study design and samples

A cross-sectional study was used, and sampling was conducted only once. Of four poultry multiplication centers in SNNPR, two (i.e. Bonga and Hawassa centers) were randomly selected. White leg horn breed was reared at both Bonga and Hawassa multiplication centers. Bonga Poultry Multiplication Center (BPMC) is mandated mainly to distribute improved commercial chickens to zones in the south and western part of the region, such as Kaffa, Shaka and Bench Maji zones. Hawassa Poultry Multiplication Center (HPMC) serves zones in the northeastern of the region. In addition, one commercial farm for Bovans brown breed was included from Hawassa for comparison. The Bonga center had three houses for layer and eight houses for growers. HPMC had 12 houses for layers and 4 houses for growers and chicks. Hawassa commercial farm had houses for layers alone. These multiplication centers disseminate growers and chicks of white leghorn and brown Bovans breeds for producers and farmers. The average number of chickens per house was 1000.

The minimum required sample size was calculated based on an expected prevalence of 15.4% [23] with 95% desired confidence interval and 5% absolute precision using the formula described in Thrusfield et al. [29]. Accordingly, 270 chickens (100 from BPMC, 90 from HPMC and 80 from Hawassa commercial farm) were sampled. In addition, environmental (bedding) samples were collected from these farms. Hand swabs of six personnel working in the poultry houses were sampled from Hawassa multiplication center and three from Bonga. The personnel were healthy and had no symptoms of infection. Human samples were collected based on an ethical approval (Ref. RD/LT/194/2013) and sampled based on their consent. The aim of hand swabbing was to evaluate the hygiene and biosecurity practices of workers in order to use the results as a risk indicator for the development of zoonoses and involvement of the workers in the dissemination of the pathogens on the farm(s). Along with the hand swab samples, farm attendants were interviewed in relation to the routine management practices of the multiplication centers and farm that could be risk factors for salmonellosis.

### Sampling and sample transportation

A sterile cotton tipped swab (2 × 3 cm) was first soaked in approximately 10 mL buffered peptone water (BPW, Oxoid Ltd., England). Then cloacal/fecal samples were collected using the pre-moistened swab. In addition, both sides of the right and left hands of farm attendants were rubbed using pre-soaked swab. Each swab sample was placed in a separate universal bottle with 4 mL BPW. The wooden shaft was broken off; and the cotton swab was left inside the bottle. The swab samples were taken according to the method described in ISO-17604 (2003) [30]. The collected samples were transported in icebox to the Laboratory of Microbiology in College of Veterinary Medicine and Agriculture, Addis Ababa University.

### Bacteriological isolation and identification

The isolation was performed using the conventional bacteriological methods for the detection of *Salmonella* standard guidelines [31]. The bacteriological culture media and biochemical tests were prepared according to manufacturer’s recommendations.

#### Non-selective pre-enrichment

The swab samples were pre-enriched in BPW in the ratio of one to nine (i.e. 25 mL of the sample was inoculated into 225 mL of BPW) and incubated at 37 °C for 24 h.

#### Selective enrichment

Rappaport-Vassiliadis medium (RV, Oxoid Ltd., England) broth and Müller Kauffman Tetrathionate (MKTT, Oxoid Ltd., England) broth were used for selective enrichment of the samples. From the pre-enriched sample, 0.1 mL was transferred into a tube containing 10 mL of RV broth and incubated at 42 °C for 24 h. Another 1 mL was transferred into a tube containing 10 mL of MKTT broth and incubated at 37 °C for 24 h.

#### Plating out and identification

Xylose lysine desoxycholate (XLD, Oxoid Ltd., England) agar and brilliant green agar (BGA, Oxoid Ltd., England) plates were used for plating out and identification. A loop full of inoculums from each RV and MKTT broth cultures were plated onto XLD and BGA plates and incubated at 37 °C for 24 h. After incubation, the plates were examined for the presence of typical and suspect *Salmonella* colonies. Typical colonies of *Salmonella* grown on XLD agar have a black center and a lightly transparent zone of reddish color due to the color change of the media [31] while hydrogen sulfide-negative variants grown on XLD agar are pink with a darker pink center. Lactose-positive *Salmonella* grown on XLD agar are yellow with or without blackening. Typical colonies of *Salmonella* on BGA are pink, 1 mm to 2 mm in diameter, and change the color of medium to red.

#### Biochemical tests

For the confirmation of the typical *Salmonella* colonies using biochemical tests, typical and suspected colonies of *Salmonella* were selected from the selective plating media, streaked onto the surface of nutrient agar (Oxoid Ltd., England) plates and incubated at 37 °C for 24 h according to ISO 6579 [31]. All suspected non-lactose fermenting *Salmonella* colonies were picked from the nutrient agar and inoculated into triple sugar iron (TSI) agar, Simmon’s citrate agar, urea broth, tryptone water, methylene red and Voges-Proskauer (MR-VP) broth (Oxoid Ltd., England) and incubated for 24 at 37 °C. A colony was considered *Salmonella* if an alkaline slant (Red) with acid butt (yellow) on TSI with hydrogen sulfide was produced, positive for lysine (purple), negative for urea hydrolysis (red), negative for tryptophan utilization (indole test) (yellow-brown ring) and negative for Voges-Proskauer (colorless) [32].

### Antimicrobial susceptibility testing

The antimicrobial susceptibility testing was performed using the disc diffusion method. The result was interpreted as per the recommendation of National Committee for Clinical Laboratory Standards Institute (CLSI) [33]. In short, isolated colonies from the nutrient agar plates were transferred into tubes containing 5 mL of tryptone soya broth (Oxoid Ltd., England). The broth culture was incubated at 37 °C for 4 h until it achieved 0.5 McFarland turbidity standards. A sterile cotton swab was used to swab the inoculum uniformly over the surface of Muller Hinton agar plate. The plates were held at room temperature for 30 min to allow drying and tested for ampicillin (AMP) 10 μg, gentamicin (CN) 10 μg, kanamycin (K) 30 μg, ciprofloxacin (CIP) 5 μg, chloramphenicol (C) 30 μg, sulfamethoxazole-trimethoprim (SXT) 25 μg, tetracycline (TE) 30 μg, nalidixic acid (NA) 30 μg, streptomycin (S) 10 μg, and cefoxitin (FOX) 30 μg. The plates were incubated at 37 °C for 24 h. The diameter of the zones of inhibition was classified as resistant, intermediate, or susceptible according to the CLSI [33]. All antibiotics were Oxoid Ltd., England made and the expiry dates were 2018.

### Data management and analysis

Data of laboratory results were entered in Microsoft Excel spread sheet and analyzed using SPSS version 20. Descriptive analysis was conducted to determine the prevalence of *Salmonella* in the centers and the farm. Association of risk factors with *Salmonella* isolation frequencies was analyzed using χ^2^ test (Fisher’s exact test). Furthermore, univariate and multivariate logistic regression analyses were performed to reveal the strength of association of the potential risk factors and *Salmonella* prevalence. The significance level was set at α = 0.05.

## Results

### Substandard biosecurity and personnel hygiene practices in the centers

Interviews with center supervisors revealed that the management practices at the breeding and multiplication centers were generally poor as summarized in Table 1. The hygiene of the poultry houses was also sub-standard, as litter changes and pest control were not carried out regularly. Working and protective materials were commonly used between houses. Personnel engaged in poor safety practices (i.e. hand washing, etc.). Antibiotics were prescribed irrationally based on symptoms without diagnosing the causative agent for the sick animals on the farms. In this regard, suspicion of a single sick chicken in the centers often led to blanket prescription of antimicrobials to all the chickens in the centers. Over prescription of drugs was practiced to avoid the risk of the undetected spread of pathogens and onset of disease outbreak at the centers (Table 1).

**Table 1:** Responses of supervising managers to interview about routine poultry farm practices, particularly on factors associated to *Salmonella* emergence, maintenance and spread

No.	Risk factors related to *Salmonella* emergence, maintenance and spread	Bonga center	Hawassa center	Hawassa commercial farm
1	Litter change when new flock introduced to the house	No	No	No
2	Poultry-house disinfection before placement	Yes	Yes	Yes
3	Presence of pests in the houses (cockroaches, rats, etc.)	Yes	Yes	No
4	Pest control	No	No	No
5	Presence of other animal species in the farm	No	No	No
6	Farm access to wild birds	Yes	Yes	Yes
7	Boots for workers	Yes	Yes	Yes
8	Common use of working and protective materials between houses	Yes	Yes	Yes
9	Always boots dipping before entering poultry houses	No	No	No
10	Free access to visitors and other workers of the farm	Yes	Yes	Yes
11	Source of water for chickens	Well	Well	Pipe
12	Conservation of sick chickens together with layer house	Yes	Yes	Yes
13	Storage of manure inside the farm compound	Yes	Yes	Yes
14	Antibiotic use without identification of disease etiology	Yes	Yes	Yes
15	Poultry management types	Litter	Litter	Cage
16	Washing of hands before handling chickens	No	No	No
17	Regular cleaning of watering and feeding trough	No	No	No
18	All in-all out practice	No	No	No
19	Disposal of a dead chicken in the compound	Yes	Yes	No
20	Rearing of different age groups of chickens in the farm	Yes	Yes	No
21	Regular health checkup for workers	No	No	No
22	Recent history of workers having signs of diarrhea	Yes	Yes	No

### Prevalence of *Salmonella* distribution

*Salmonella* was isolated from 45 of the 270 samples (16.67%); 36 of 244 cloacal swabs (14.8%), 6 of 17 pooled bedding sample (35.3%), and 3 of 9 hand swabs sampled from farm attendants (33.3%). Both bedding and hand swab samples had significantly (*p* = 0.028) higher *Salmonella* isolation rate than cloacal samples. The prevalence of *Salmonella* was significantly higher at Bonga than at Hawassa (*p* = 0.000). The Bonga poultry multiplication center had a significantly higher prevalence of *Salmonella* than the other two multiplication centers (*p* = 0.001). Cage versus litter nor Leghorn versus Bovans did not significantly (*p* > 0.05) affect the prevalence of *Salmonella* detected (Table 2). Young age groups (chicks and cocks) had a significantly (*p* = 0.003) higher prevalence followed by layers than the other age groups.

**Table 2:** Prevalence of *Salmonella* in chickens, farm attendants and bedding in Hawassa and Bonga chicken breeding and multiplication centers in Southern Ethiopia

Risk Factors		# sample	Positive	Prevalence (%)	*p*-value
Study area	Bonga	100	27	27	0.000
	Hawassa	170	18	10.6	
Farms	Bonga multiplication center	100	27	27	0.001
	Hawassa multiplication center	80	11	13.8	
	Hawassa commercial farm 2	90	7	7.8	
Sample type	Bedding (environment)	17	6	35.3	0.028
	Hand swab (personnel)	9	3	33.3	
	Cloacal swab (chickens)	244	36	14.8	
Housing	Litter	190	34	17.9	0.404
	Cage	80	11	13.9	
Breed	White leghorn	190	34	17.9	0.404
	Bovans	80	11	13.8	
Age group	Chicks	21	10	47.6	0.003
	Cock	19	4	21.1	
	Cockerel	3	0	0	
	Layer	194	29	14.9	
	Pullet	33	2	6.1	
Total		270	45	16.7	

Univariate analysis of risk factors indicated that samples from Bonga had a significantly (*p* = 0.001) higher odds of being *Salmonella* positive than those from Hawassa. Study areas, multiplication centers and sample types had a significant association with *Salmonella* prevalence (Tables 2 and 3). Finally, multivariate logistic regression analysis was conducted for association between risk factors and *Salmonella* isolation. After the effect of other risk factors were adjusted for, only sample type had a significant effect on *Salmonella* recovery. In this regard, bedding had higher *Salmonella* contamination than chicken (cloacal) samples [OR for bedding vs. poultry cloacal = 7.5; 95% CI for OR = 2.0–25; *p* = 0.002].

**Table 3:** Univariate analysis of *Salmonella* infection using various risk factors in Hawassa and Bonga chicken breeding and multiplication centers in Southern Ethiopia

Risk Factors	Variables	OR	95% CI for OR	*p*-value
Study area	Bonga vs. Hawassa	3.1	1.6–6.0	0.001
Breeding center	Bonga vs. Hawassa	2.3	1.1–5.0	0.033
Sample source	Bedding vs. Chicken	8.3	2.5–25.0	0.000

### Antimicrobial susceptibility test

Forty-five *Salmonella* isolates were subjected to the antimicrobial susceptibility test. All of the 45 isolates (100%) were resistance to kanamycin and sulfamethoxazole-trimethoprim. Almost all (44 isolates, 97.8%) also had resistance to ampicillin, cefoxitin, nalidixic acid, streptomycin, and tetracycline. Resistance to chloramphenicol and ciprofloxacin was 91.1% and 31.1%, respectively. All isolates (100%) were susceptible to gentamicin with three isolates manifested intermediate breakpoints (Table 4).

**Table 4:**
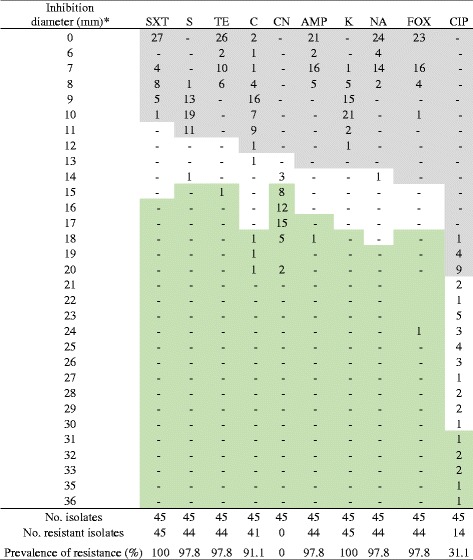
Inhibition ranges and number of resistant, intermediate and susceptible isolates of *Salmonella* to 10 antimicrobials tested as per the CLSI (2012) [34]

*AMP* ampicillin; *C* chloramphenicol; *CN* gentamicin; *CIP* ciprofloxacin; *FOX* cefoxitin; *K* kanamycin; *NA* nalidixic acid; *S* streptomycin; *SXT* sulfamethoxazole-trimethoprim; *TE* tetracycline

*The diameter indicated zone of inhibition of the antimicrobials during disc diffusion test (mm). The top dark shaded part within the table indicated breakpoints for resistance along with the frequency of isolates. The middle unshaded part indicated breakpoints for intermediate and the lower green shaded part indicated susceptible isolates

#### Phenotypic patterns to antimicrobial resistance

All 45 isolates (100%) were multi-drug resistant to ≥3 antimicrobials. Forty-two isolates (93.3%) exhibited resistance to ≥8 antimicrobials simultaneously (Table 5). The 45 *Salmonella* isolates manifested nine different phenotypic patterns to 10 antimicrobials. The most widely distributed pattern was resistance to AMP*C*CIP-I*FOX*K*NA*S*SXT*TE with 24 (53.3%) of the 45 isolates exhibiting this pattern. Nine of the 45 isolates (20.0%) displayed the AMP*C*CIP*FOX*K*NA*S*SXT*TE pattern.

**Table 5:** Lists of AMR patterns of *Salmonella* isolates from chicken breeding and multiplication centers in response to 10 antimicrobials in Southern Ethiopia

The nine AMR pattern	Number antimicrobials resistance	Number of isolates	Prevalence (%)
C*K*S-I ^a^*SXT	3(+S-1)	1	2.2
AMP*C-I*FOX*K*NA*S*SXT*TE	7(+C-I)	1	2.2
AMP*CIP-I*FOX*K*NA*S*SXT*TE	7(+CIP-I)	1	2.2
AMP*C*FOX*K*NA*S*SXT*TE	8	4	8.9
AMP*CIP*FOX*K*NA*S*SXT*TE	8	2	4.4
AMP*C*CIP-I*FOX*K*NA*S*SXT*TE	8(+CIP-I)	24	53.3
AMP*C*CN-I*CIP-I*FOX*K*NA*S*SXT*TE	8(+CN-I*CIP-I)	1	2.2
AMP*C*CIP*FOX*K*NA*S*SXT*TE	9	9	20.0
AMP*C*CN-I*CIP*FOX*K*NA*S*SXT*TE	9(+CN-I)	2	4.4
		45	100.0

*AMP* ampicillin; *AMR* antimicrobial resistance; *C* chloramphenicol; *CN* gentamicin; *CIP* ciprofloxacin; *FOX* cefoxitin; *K* kanamycin; *NA* nalidixic acid; *S* streptomycin; *SXT* sulfamethoxazole-trimethoprim; *TE* tetracycline

^a^I indicates intermediate

Three *Salmonella* isolates from hand swabs displayed disturbing AMR to 8 antimicrobials (two isolates) and 9 antimicrobials (one isolate). All isolates from personnel and poultry bedding were resistant to at least eight antimicrobials, but susceptible to gentamicin and ciprofloxacin (Table 6). The six isolates from bedding samples revealed resistant to eight or nine antimicrobials (four and two isolates respectively). The distribution of resistance patterns of the isolates across different locations, management, breed and sample type in southern Ethiopia is indicated in Table 6.

**Table 6:** Antimicrobial resistance patterns of *Salmonella* isolates collected from chicken multiplication centers of different locations, management, breed and sample type in Southern Ethiopia

AMR pattern (No. of drugs showing resistant + intermediate)	Study area (*N* = 45)			Management type			Breed type			Sample type		
		*n* ^a^	%		*n*	%		*n*	%		*n*	%
AMP*C-I^¶^*FOX*K*NA*S*SXT*TE (7 +^¶^1)	Hawassa	1	2.2	Litter	1	2.2	WLH	1	2.2	Cloacal swab	1	2.2
AMP*C*CIP-I*FOX*K*NA*S*SXT*TE (8 + 1)	Bonga	10	22.2	Cage	8	17.8	Bovans	8	17.8	Cloacal swab	18	40.0
	Hawassa	14	31.1	Litter	16	35.6	WLH	16	35.6	Hand swab	2	4.4
										Bedding	4	8.9
	Total	24	53.3	Total	24	53.3	Total	24	53.3	Total	24	53.3
AMP*C*CIP*FOX*K*NA*S*SXT*TE (9)	Bonga	8	17.8	Cage	1	2.2	Bovans	1	2.2	Cloacal swab	8	17.8
	Hawassa	1	2.2	Litter	8	17.8	WLH	8	17.8	Bedding	1	2.2
	Total	9	20.0	Total	9	20.0	Total	9	20.0	Total	9	20.0
AMP*C*CN-I*CIP-I*FOX*K*NA*S*SXT*TE (8 + 2)	Bonga	1	2.2	Litter	1	2.2	WLH	1	2.2	Cloacal swab	1	2.2
AMP*C*CN-I*CIP*FOX*K*NA*S*SXT*TE (9 + 1)	Bonga	2	4.4	Litter	2	4.4	WLH	2	4.4	Hand swab	1	2.2
										Bedding	1	2.2
										Total	2	4.4
AMP*C*FOX*K*NA*S*SXT*TE (8)	Bonga	3	6.7	Cage	1	2.2	Bovans	1	2.2	Cloacal swab	4	8.9
	Hawassa	1	2.2	Litter	3	6.7	WLH	3	6.7			
	Total	4	8.9	Total	4	8.9	Total	4	8.9			
AMP*CIP-I*FOX*K*NA*S*SXT*TE (7 + 1)	Bonga	1	2.2	Litter	1	2.2	WLH	1	2.2	Cloacal swab	1	2.2
AMP*CIP*FOX*K*NA*S*SXT*TE (8)	Bonga	1	2.2	Cage	1	2.2	Bovans	1	2.2	Cloacal swab	2	4.4
	Hawassa	1	2.2	Litter	1	2.2	WLH	1	2.2			
	Total	2	4.4	Total	2	4.4	Total	2	4.4			
C*K*S-I*SXT (3 + 1)	Bonga	1	2.2	Litter	1	2.2	WLH	1	2.2	Cloacal swab	1	2.2

#Number of isolates exhibiting the indicated resistance pattern in the respective rows. The “(+)” in the first column indicated number of drug types that manifested intermediate response category to the isolates. The number of isolates tested for AMR from Hawassa (*n* = 18), Bonga (*n* = 27), Cage (*n* = 11), Litter (*n* = 34), Bovans Brown (*n* = 11), White Leghorn (WLH) (*n* = 34), Cloacal swab (*n* = 36), bedding (*n* = 6) and personnel hand swab (*n* = 3). AMP, ampicillin; AMR, antimicrobial resistance; C, chloramphenicol; CN, gentamicin; CIP, ciprofloxacin; FOX, cefoxitin; K, kanamycin; NA, nalidixic acid; S, streptomycin; SXT, sulfamethoxazole-trimethoprim; TE, tetracycline

^¶^Indicates intermediate breakpoints

## Discussion

*Salmonella* is one of the important pathogens in the poultry industry [34]. Additionally, it is one of the major cause of bacterial foodborne gastroenteritis of humans with poultry being indicated as the major source for human infection [17, 35]. *Salmonella* can enter the food supply at many points in the food production chain (processing, distribution, preparation, or handling steps). Thus, food safety should involve intervention at all of the steps along the production chain in order to safeguard the health of consumers [36]. Some intervention have been successful against *Salmonella* in the processing plants [37]. The effect of *Salmonella* intervention on the farms to reduce *Salmonella* prevalence along the food chain requires further evaluation. Understanding of the ecology of *Salmonella* helps to reduce horizontal and vertical transmission. Furthermore, tracing the origin of *Salmonella* outbreaks and the antimicrobial resistant patterns of isolates are fundamental to containing the risk. However, little is known about the *Salmonella* problem in chicken breeding, multiplication and distribution centers in Ethiopia. The present study was conducted to determine the prevalence of *Salmonella* and their AMR in poultry production and multiplication centers in southern Ethiopia.

The overall prevalence of *Salmonella* was 16.7% in this study. Our finding is similar to the 17.9% prevalence on layer farms in Italy [38], but lower than the 42.7% reported from Jimma in western Ethiopia [21] and 36.1% in broilers in Japan [39]. The variation in prevalence reported could be due to the variation in the sample type, location, breed, and/or age of the chickens [40]. Given that observation of a single sick chicken can led to a blanket prescription of antimicrobials to the entire flock at the centers (as per the respondents), the intensive antimicrobial use currently could also contribute to the lowered prevalence in our findings. The present finding was higher than the 8% prevalence reported from small-scale chicken farms from Hawassa [41]. It has been reported that farm type and chicken flock density might have a significant impact on the prevalence of *Salmonella*, particularly about 7 times higher on large-scale versus small-scale farms [42] and nearly 8 times higher on conventional compared to organic farms [43].

In the current study, breed, age, sample type and location have been evaluated as triggering factors for *Salmonella* occurrence. In this regard, both White Leghorn and Bovans Brown chicken breeds were equally susceptible. However, some researchers have reported that breed differences on the level of susceptibility to *Salmonella* exists among different chicken lines [24, 44]. In this regard, resistant lines to *S. Typhimurium* have also shown resistance to *S. Gallinarum*, *S. Pullorum*, and *S. Enteritidis*, and lines susceptible to *S. Typhimurium* are more susceptible to the other *Salmonella* serotypes [44]. This suggests that a general mechanism of resistance that may apply to all serotypes of *Salmonella* in chicken [44]. Three levels of susceptibly have been noticed among chicken lines to *Salmonella*. These include (i) highly susceptible chicken lines which reveal greater localization of *Salmonella* in the reproductive tract (vertical transmission) in addition to harboring *Salmonella* in other body parts, (ii) moderately resistant which reveal greater localization of *Salmonella* in the intestine along with long term persistence in the liver and spleen, and (iii) highly *Salmonella* resistant chicken lines [45]. As prophylactic measures, vaccination and use of antibiotics are insufficient to eradicate salmonellosis in poultry stocks, some researchers have tried to control *Salmonella* infection, its carrier-state and drug resistant *Salmonella* strains through rearing *Salmonella* resistant chicken lines [46].

Young age groups (chicks and cock) harbored higher *Salmonella* prevalence in this study. This suggests a higher susceptibility of younger chicks to *Salmonella*. In this regard, it has been reported that the ability to survive infection increases with age, and mortality is greatest in newly hatched chicks [47]. Unfortunately, once *Salmonella* infects chicks at a young age, they remain in a carrier state for a prolonged period of time [48]. The high susceptibility and inability of the young chicks to clear the infection (persistent carrier) could be due to the fact that infection at a younger age is less effective in producing protective immunity than infection in older chickens [48]. Chicks infected at 4-days of age remain (i) a carrier until they start lay eggs, (ii) produced *Salmonella* infected eggs; and hence, (iii) the progeny is infected via vertical transmission [45]. Following young chicks, laying hens were the next most susceptible age group in this study. The higher prevalence of *Salmonella* infection in laying hens might be due to physiological stress of egg production and molting, which significantly depresses the immune response and increases the susceptibility to *Salmonella* infection [49]. The interesting part of *Salmonella* is its ability to infect chicks at early age, persist for long time in the infected chickens and allows for vertical transmission to the progeny through contaminated eggs. This may complicate the epidemiology and control of *Salmonella* in chicken multiplication centers of Ethiopia, as *Salmonella* was highly abundant among young chicks followed by laying hens in this study.

The abundance of *Salmonella* distribution was higher in Bonga (27%) than Hawassa (10.6%) area in the present study. Significantly more isolates were detected in Bonga than Hawassa (OR = 3.1). Bonga has a humid climate with diverse fauna (cockroaches, mice, birds and other animals), which is important for persistence and spread of *Salmonella*. These factors might have contributed to the higher abundance of *Salmonella* at Bonga than at Hawassa. Furthermore, the higher tendency of rearing of mixed age groups in the Bonga area than in the Hawassa area may also contribute to the current difference in prevalence. In this regard, multi-age management of layers as opposed to all-in/all-out practice has been associated with a higher risk of *Salmonella* contamination [38]. The management practice of single age rearing is reported to protect of chickens from several infectious diseases as opposed to mixed age chicken rearing [50]. Differences in biosecurity practices and management standards on the poultry farms may explain this variation. It has been reported that poor biosecurity exposes the birds to numerous potential sources of *Salmonella* contamination [16]. Furthermore, the risk of *Salmonella* contamination in flocks reared on litter was higher with multiage management than on farms with an all-in/all out practice or farms with a single flock [50]. However, cage and litter based chicken rearing system didn’t have a significant difference in this study.

*Salmonella* were recovered from a significant number of poultry bedding samples (environment, 35.3%) in our study. Our finding is lower than the 58.8% reported from poultry bedding in Nigeria [51]. The difference may be due to the environmental persistence of *Salmonella* which affects its epidemiology in poultry by creating opportunities for the horizontal transmission of *Salmonella* among chickens within the flocks [52] and *Salmonella* can be introduced into the flocks from many different sources [25, 26]. There are a lot of possible sources of *Salmonella* contamination in poultry flocks from cockroaches and lesser mealworms which can carry *Salmonella* internally and externally and spread *Salmonella* throughout the flock [53, 54]. Mice have also been important vectors for *Salmonella* in laying hens [55]. Practices such as unhygienic farming activities, lack of adequate biosecurity measures and movement of birds and equipment from one house or farm to another often worsens the situation. Mice, wild birds, ants and other insects have been shown to be important agents for *Salmonella* transmission among chickens, flocks and farms [56].

Currently, 33.3% of the 9 attendants carried *Salmonella* on their hands contributing three isolates (6.7%) of the 45 *Salmonella* isolates collected in this study. Personnel hand contamination is a real concern due to limited supply of personnel hygiene supplies, poor hand washing practices and the high levels of *Salmonella* in the beddings of this study. These factors could lead to (i) self-infection of personnel from the *Salmonella* on their hands and possible contamination of their families, (ii) spread of infection to individuals in the centers and guests by routine hand shaking as part of the cultural greetings in Ethiopia and (iii) spreading infection between poultry houses in the centers. Prompt attention is necessary to address the contamination of personnel hands in order to safeguard the safety of the personnel and the animals on the farms. *Salmonella* is responsible for 1.4 million cases of human illness, 20,000 hospitalizations and over 500 deaths annually in US [17] of which 46.4% of the isolates are from animal sources mainly poultry [35]. Of concern in this study is that all of the isolates from hands were multidrug resistant.

Fifty percent of the isolates in this study were resistant to at least one antimicrobial. This is in agreement with the 57–66% of the isolates in Europe [57] and 79.8% of isolates from processed poultry in US [18]. Another study of a national monitoring system reported AMR of 30.5% among the total US isolates [58].

In this study, the most effective drug was gentamicin with 0/45 (0%) isolates resistant, followed by ciprofloxacin with 14/45 (31.1%) were resistant. No reports of resistance to gentamycin or ciprofloxacin have been made in sheep and goats isolates in central Ethiopia [59], dairy farm isolates in Addis Ababa [60] or human isolates in Nigeria [61]. Gentamycin is still effective against *Salmonella* irrespective of the time or location of the study. We have not found information on whether gentamycin is or is not widely used, prescribed or distributed in Ethiopia or other African countries. Lack of use of these two antimicrobials could explain the lack of resistance in the recovered isolates. In two previous studies conducted in Ethiopia, 41.2 and 46% of isolates were resistant to tetracycline; 22.5 and 75% were resistant to streptomycin; and 30% were resistant to chloramphenicol. In our study, 100% of isolates were resistant to kanamycin and suflamethoxazole-trimethoprim; 97.8% of isolates were resistant to ampicillin, cefoxitin, nalidixic acid, streptomycin and tetracycline; and 91.1% of the isolates were resistant to chloramphenicol. These findings represent a potential increase in the antimicrobial resistance in Ethiopian flocks. This increase could be due to the unregulated increase in the use of prescription antimicrobials [14], often purchased and used by unskilled practitioners in the veterinary and public health sectors [62]. There is a lack of compliance and monitoring for antimicrobials at all levels in Ethiopia. Additionally, the use of antimicrobial drugs at sub-therapeutic or prophylactic dosages in food animals may promote on farm selection of antimicrobial resistance genes in *Salmonella* as well as other potential human and animal pathogens.

Overall, 42 isolates (93.4%) exhibited a MDR phenotype to more than 8 antimicrobials simultaneously. These MDR isolates had different resistant pattern. Specifically, 11 isolates were resistant to 9 antimicrobials and 31 were resistant to 8 antimicrobials while two were resistant to only 7 antimicrobials and one isolate was resistant to only three antimicrobials. This level of MDR was higher than the 32.65% [63], 44.8% [64], and 83.3% [60] reported previously in Ethiopia. Interestingly, the number of MDR *Salmonella* isolates has increased in many countries. In the US, 53.4% isolates from processed poultry are MDR [18] and 15–18% of the European isolates [57] are MDR. The widespread resistance of *Salmonella* isolates in this study may suggest that the gross indiscriminate use of antimicrobial agents in the poultry production setting is responsible for this increase, which has also been suggested by other researchers [65, 66]. In fact, multiplication centers indiscriminately prescribe drugs for the entire flock if ill health is noticed in a single chicken on the ground in order to prevent the spread of infection to healthy chickens at the centers. This excessive misuse of antimicrobial drugs subjects all bacteria present to selective pressures and could lead to the development of resistance to the drugs currently in use and proposed for future use [67]. The incorporation of antibiotics in the animal diets at sub-therapeutic levels to serve as a prophylaxes and/or a growth promotants has invariably contributed to the development of antibiotic resistant strains of *Salmonella* [68]. Overall, abundance the abundance MDR *Salmonella* isolates from poultry multiplication centers render poultry meat and eggs a very important potential source of resistant bacteria and hence a serious potential public health concern. However, the limitation of lack of serotype and genotype data in this study precludes the analysis of the relatedness of the specimens collected from different centers including from humans.

## Conclusions

Of 270 samples tested, *Salmonella* was isolated from 45 (16.7%) of the samples. *Salmonella* was recovered from chicken cloaca, the environment (bedding) and human hands in the multiplication centers. Its distribution was associated with location of the individual center/farm, age of the chickens and the sample type indicating these factors are important potential factors for intervention. Substandard management practices and poor biosecurity exposes the birds to numerous potential sources of *Salmonella*. Overall, 93.4% of the 45 isolates exhibited resistance against ≥8 antimicrobials while two isolates were resistant to 7 antimicrobials and one was resistant to three antimicrobials. This implies that all isolates are MDR (100%). The high percentage of MDR isolates recovered indicate the potential importance of chickens as a source of MDR *Salmonella* infections and a serious public health concern, because the multiplication centers rely on the over use of drugs while practicing poor biosecurity and sub-standard management. We conclude that the poultry breeding, multiplication and distribution centers in Ethiopia, as they currently stand, are a source for the dissemination of pathogens and drug resistant pathogens, at least *Salmonella*. Therefore, we recommend personnel training on personal hygiene, biosafety and farm biosecurity for all farm attendants and visitors; education on zoonotic pathogens; restrictions on the uncontrolled use of antimicrobials; and establishment of a standard pathogen and AMR monitoring system in poultry production. Overall, we hypothesize that control of *Salmonella* infections in poultry will be one of the important steps to control *Salmonella* infections in public health.

## Abbreviations

AMP: ampicillin
AMR: antimicrobial resistance
BPMC: Bonga poultry multiplication center
C: chloramphenicol
CIP: ciprofloxacin
CLSI: Clinical laboratory Standards Institute
CN: gentamicin
FOX: cefoxitin
HPMC: Hawassa poultry multiplication center
K: kanamycin
MDR: Multi-drug resistance
NA: nalidixic acid
S: streptomycin
SNNPR: Southern Nations, Nationalities and Peoples Region
SXT: sulfamethoxazole-trimethoprim
TE: tetracycline

## References

[1] Duguma R. Understanding the role of indigenous chicken during the long walk to food security in Ethiopia. Livestock Res Rural Dev*.* 2009;21(116). http://www.lrrd.org/lrrd21/8/dugu21116.htm. Accessed 07 Feb 2017.

[2] CSA (Central Statistical Agency). Agricultural Sample Survey 2014/15 [2007 E.C.]. Volume II report on livestock and livestock characteristics (private peasant holdings). Addis Ababa; 2015.

[3] Boere a, Vernooij a, duns H, Legesse M, Kidane D. Business opportunities report poultry #3 in the series written for the Ethiopian Netherlands business event 5–6. Rijswijk, The Netherlands. November 2015:2015.

[4] Duguma R. Phenotypic characterization of some indigenous chicken ecotypes of Ethiopia. Livestock Res Rural Dev. 2006;18(131). http://www.lrrd.org/lrrd18/9/dugu18131.htm. Accessed 07 Feb 2017.

[5] Wiggins LL (1958). IECA and JATS staff report. Agriculture of Ethiopia.

[6] Herduck J. IECA and JATS staff report. Agriculture of Ethiopia. 1961;8.pp.

[7] Pagani P, Wossene A. Review of the new features of the Ethiopian poultry sector biosecurity implications. Food and Agriculture Organization of the United Nations. FAO. 2008;29. http://www.fao.org/docrep/013/al837e/al837e00.pdf Accessed 08 Feb 2017.

[8] Ethiopia Livestock Master Plan (ELMP). Roadmaps for growth and transformation. A contribution to the Growth and Transformation Plan II (2015–2020). ILRI Editorial and Publishing Services. Addis Ababa, Ethiopia; 2015.

[9] Besbes B, Ducrocq V, Foulley JL, Protais M, Tavernier A, Tixierboichard M (1993). Box-cox transformation of egg-production traits of laying hens to improve genetic parameter estimation and breeding evaluation. Livestock Prod Sci.

[10] Dana N, Duguma R, Teklewold H, Aliye S. Transforming village poultry systems into small agro-business ventures: a partnership model for the transfer of livestock technologies in Ethiopia. Livestock Res Rural Dev. 2006;18(12). http://www.lrrd.org/lrrd18/12/dana18169.htm. Accessed 08 February 2017.

[11] Reta D, Negussie D, Alemu Y. Comparative production performance of two exotic chicken breeds under two different feed regimes in three agro-ecologies of central Oromia, Ethiopia-a step forward for distribution or contract rearing of day old exotic chicks under rural setting. Livestock Res Rural Dev. 2012;24(9). http://www.lrrd.org/lrrd24/9/dugu24153.htm. Accessed 08 Feb 2017.

[12] Teklewold H, Dadi L, Yami A, Dana N. Determinants of adoption of poultry technology: a double hurdle approach. Livestock Res Rural Dev. 2006;18(40). http://www.lrrd.org/lrrd18/3/tekl18040.htm. Accessed 08 Feb 2017.

[13] Wossene A. Poultry bio-security study in Ethiopia. A consultancy report for Food and Agriculture Organization of the United Nations, Addis Ababa, Ethiopia. 2006;35

[14] Beyene T, Endalamaw E, Tolossa Y, Feyisa A (2015). Evaluation of rational use of veterinary drugs in Bishoftu. Central Ethiopia BMC Res Notes.

[15] Jordan FTW, Pattison M (1996). Poultry diseases.

[16] Liljebjelke K, Hofacre T, White S, Ayers S, Maurer J (2005). Vertical and horizontal transmission of *Salmonella* within integrated broiler production system. Foodborne Pathog.

[17] Mead PS, Slutsker L, Dietz V, McCaig LF, Bresee JS, Shapiro C (1999). Food-related illness and death in the United States. Emerg Infect Dis.

[18] Parveen S, Taabodi M, Schwarz JG. Oscar TP, Harter-Dennis J, White DG. Prevalence and AMR of *Salmonella* recovered from processed poultry. J Food Prot 2007;70(11):2466–2472. 10.4315/0362-028x-70.11.2466 18044422

[19] Davis MF, Price LB, Liu CMH, Silbergeld EK (2011). An ecological perspective on US industrial poultry production: the role of anthropogenic ecosystems on the emergence of drug-resistant bacteria from agricultural environments. Curr Opin Microbiol.

[20] Berhe N, Afera B, Abebe N, Tesfaya A, Kalayou S (2012). Seroprevalence of *Salmonella* pullorum infection in local and exotic commercial chicken from Mekelle areas, northern Ethiopia. Rev Electrón Vet.

[21] Kindu A, Addis M (2013). A survey on *Salmonella* infection among chicken flocks in Jimma town. Ethiopia World Appl Sci J.

[22] Molla B, Mesfin A, Alemayehu D (2003). Multiple antimicrobial-resistant *Salmonella* serotypes isolated from chicken carcass and giblets in Debre Zeit and Addis Ababa. Ethiopia Ethiop J Health Devlop.

[23] Bekele B, Ashenafi M (2010). Distribution of drug resistance among enterococci and *Salmonella* from poultry and cattle in Ethiopia. Trop Anim Health Prod.

[24] Wigley P (2004). Genetic resistance to *Salmonella* infection in domestic animals. Res Vet Sci.

[25] Kim A, Lee YJ, Kang MS, Kwag SI, Cho JK (2007). Dissemination and tracking of *Salmonella* spp. in integrated broiler operation. J Vet Sci.

[26] Sivaramalingam T, Pearl DL, McEwen SA, Ojkic D, Guerin MT (2013). A temporal study of *Salmonella* serovars from fluff samples from poultry breeder hatcheries in Ontario between 1998 and 2008. Can J Vet Res.

[27] Teshome A. Agriculture, growth and poverty reduction in Ethiopia: policy processes around the new PRSP (PASDEP). In a paper for the Future Agricultures Consortium Workshop, Institute of Development Studies, University of Sussex, UK; 2006. p. 20–22.

[28] CSA (Central Statistical Agency). Agriculture sample survey 2014/2015 (2007 E.C.) (September–January 2014/2015), Volume VII. Report on crop and livestock product utilization (Private peasant holdings, Meher Season). Addis Ababa; 2015.

[29] Thrusfield M. Veterinary Epidemiology. 2^nd^ ed. Black well, Cambridge; 2005. p. 175–187.

[30] ISO-17604. Microbiology of food and animal feeding stuffs: Carcass sampling for microbiological analysis. ISO, Geneva; 2003. p. 1–17.

[31] ISO-6579. Microbiology of food and animal feeding stuffs: Horizontal method for the detection of *Salmonella* spp*.* ISO, Geneva; 2002. p. 511–525.

[32] Quinn PJ, Carter ME, Markey B, Carter GR. *Enterobacteriaceae*. In: Clinical Veterinary Microbiology. Molsby International Limited, London; 1999. p. 226–234.

[33] CLSI (Clinical Laboratory Standards Institute). Performance Standards for Antimicrobial Susceptibility Testing; Twenty Second Informational Supplement*.* CLSI document M100-S22, Wayne PA; 2012.

[34] Cosby DE, Cox NA, Harrison MA, Wilson JL, Buhr RJ, Fedorka-Cray PJ (2015). *Salmonella* and antimicrobial resistance in broilers: a review. J Appl Poult Res.

[35] Sanchez S, Hofacre CL, Lee MD, Maurer JJ, Doyle MP (2002). Animal sources of salmonellosis in humans. J Am Vet Med Assoc.

[36] CDC (Centers for Disease Control and Prevention). The food production chain-how food gets contaminated. Foodborne Outbreaks. 2015. https://www.cdc.gov/foodsafety/outbreaks/investigating-outbreaks/production-chain.html. Accessed 07 Feb 2017.

[37] Dórea FC, Cole DJ, Hofacre C, Zamperini K, Mathis D, Doyle MP (2010). Effect of *Salmonella* vaccination of breeder chickens on contamination of broiler chicken carcasses in integrated poultry operations. App Environ Microbiol.

[38] Adeline HS, Marianne C, Sophie Le B, Franc L, Isabelle P, Sandra R (2009). Risk factors for *Salmonella* enterica subsp. enterica contamination in 519 French laying hen flocks at the end of the laying period. Prev Vet Med.

[39] Ishihara K, Takahashi T, Morioka A, Kojima A, Kijima M, Asai T, Tamura Y (2009). National surveillance of *Salmonella enterica* in food-producing animals in Japan. Acta Vet Scandi.

[40] Uyttendaele MR, Debevere JM, Lips RM, Neyts KD (1998). Prevalence of *Salmonella in* poultry carcasses and their products in Belgium. Int J Food Microbiol.

[41] Kassaye A, Lencho T, Mesele A (2010). Prevalence of *Salmonella* infection in intensive poultry farms in Hawassa and isolation of *Salmonella* species from sick and dead chickens. Ethiop Vet J.

[42] Makaya PV, Matope G, Pfukenyi DM (2012). Distribution of *Salmonella* serovars and antimicrobial susceptibility of *Salmonella enteritidis* from poultry in Zimbabwe. Avian Pathol..

[43] Alali WQ, Thakur S, Berghaus RD, Martin MP, Gebreyes WA (2010). Prevalence and distribution of *Salmonella* in organic and conventional broiler poultry farms. Foodborne Pathog Dis.

[44] Bumstead N, Barrow P. Resistance to *Salmonella* gallinarum, S. pullorum, and S. enteritidis in inbred lines of chickens. Avian Dis. 1993; p.189–193. 8452495

[45] Berchieri JA, Murphy CK, Marston K, Barrow PA (2001). Observations on the persistence and vertical transmission of *Salmonella* enterica serovars Pullorum and Gallinarum in chickens: effect of bacterial and host genetic background. Avian Pathol.

[46] Calenge F, Kaiser P, Vignal A, Beaumont C (2010). Genetic control of resistance to salmonellosis and to *Salmonella* carrier-state in fowl: a review. Genet Sel Evol.

[47] Gast RK, Beard CW (1989). Age-related changes in the persistence and pathogenicity of *Salmonella* typhimurium in chicks. Poult Sci.

[48] Beal RK, Wigley P, Powers C, Hulme SD, Barrow PA, Smith AL (2004). Age at primary infection with *Salmonella* enterica serovar typhimurium in the chicken influences persistence of infection and subsequent immunity to re-challenge. Vet Immunol Immunopathol.

[49] Holt PS (2003). Molting and *Salmonella* enterica serovar Enteritidis infection: the problem and some solutions. Poult Sci.

[50] Davies R, Breslin M (2001). Environmental contamination and detection of *Salmonella* enterica serovar enteritidis in laying flocks. Vet Rec..

[51] Yhiler NY, Bassey BE (2015). Antimicrobial susceptibility patterns of *Salmonella* species from sources in poultry production settings in Calabar, Cross River state. Nigeria Am J Health Res.

[52] McIlroy SG, McCracken RM, Neilland SD, O’brien JJ (1989). Control, prevention and eradication of *Salmonella* enteritidis infection in broiler and broiler breeder flocks. Vet Rec.

[53] McAllister JC, Steelman CD, Skeeles JK (1994). Reservoir competence of the lesser worm (Coleoptera: Tenebrionidae) for *Salmonella typhimurium* (Eubacteriales: Enterobacteriaceae). J Med Entomol.

[54] Kopanic RJ, Sheldon BW, Wright CG (1994). Cockroaches as vectors of *Salmonella*: laboratory and field trials. J Food Prot.

[55] Henzler DJ, Opitz HM (1992). The role of mice in the epizootiology of *Salmonella* enteritidis infection on chicken layer farms. Avian Dis.

[56] Carrique-Mas JJ (2009). Breslin M, snow L, McLaren I, Sayers, Davies RH. Persistence and clearance of different *Salmonella* serovars in buildings housing laying hens. Epidemiol Infect.

[57] Meakins S, Fisher IS, Berghold C, Gerner-Smidt P, Tschäpe H, Cormican M (2008). 2008. Antimicrobial drug resistance in human nontyphoidal *Salmonella* isolates in Europe 2000-2004: a report from the enter-net international surveillance network. Microb Drug Resist.

[58] Crump JA, Medalla FM, Joyce KW, Krueger AL, Hoekstra RM, Whichard JM (2011). Antimicrobial resistance among invasive nontyphoidal *Salmonella* enterica in the United States, National Antimicrobial Resistance Monitoring System, 1996-2007. Antimicrob Agents Chemother.

[59] Molla W, Molla B, Alemayehu D, Muckle A, Cole L, Wilkie E (2006). Occurrence and antimicrobial resistance of *Salmonella* serovars in apparently healthy slaughtered sheep and goats of central Ethiopia. Trop Anim Health Prod.

[60] Zelalem A, Nigatu K, Zufan W, Haile G, Alehegne Y, Tesfu K (2011). Prevalence and antimicrobial resistance of *Salmonella* isolated from lactating cows and in contact humans in dairy farms of Addis Ababa. BMC Infect Dis.

[61] Akinyemi KO, Iwalokun BA, Foli F, Oshodi K, Coker AO (2011). Prevalence of multiple drug resistance and screening of enterotoxin (stn) gene in *Salmonella* enterica serovars from water sources in Lagos. Nigeria Public Health.

[62] Okeke IN, Laxminarayan R, Bhutta ZA, Duse AG, Jenkins P, O’Brien TF (2005). Antimicrobial resistance in developing countries. Part I: recent trends and current status. Lancet Infect Dis.

[63] Zewdu E, Cornelius P (2009). Antimicrobial resistance pattern of *Salmonella* serotypes isolated from food items and personnel in Addis Ababa. Ethiopia Trop Anim Health Prod.

[64] Molla B, Mohammed A, Salah W (2004). *Salmonella* prevalence and distribution of serotypes in apparently healthy slaughtered camels (Camelues dromedaries) in Ethiopia. Trop Anim Health Prod.

[65] Zhao S, Datta AR, Ayers S, Friedman S, Walker RD, White DG (2003). Antimicrobial-resistant *Salmonella* serovars isolated from imported foods. Int J Food Microbiol.

[66] Angulo FJ, Nargund VN, Chiller TC (2004). Evidence of an association between use of antimicrobial agents in food animals and anti-microbial resistance among bacteria isolated from humans and the human health consequences of such resistance. J Vet Med.

[67] Levy SB. Antibiotic resistance: an ecological imbalance. In*: Antibiotic Resistance: Origin, Evolution, Selection and Spread.* Ciba Foundation Symposium 207, Wiley, Chichester; 1997. p.1–14. 10.1002/9780470515358.ch1 9189631

[68] Shah AH, Korejo NA (2012). Antimicrobial resistance profile of *Salmonella* serovars isolated from chicken meat. J Vet Anim Sci.

